# Resveratrol enhances ionizing radiation-induced premature senescence in lung cancer cells

**DOI:** 10.3892/ijo.2013.2141

**Published:** 2013-10-17

**Authors:** HONGMEI LUO, LU WANG, BRADLEY A. SCHULTE, AIMIN YANG, SHENGSONG TANG, GAVIN Y. WANG

**Affiliations:** 1 Department of Pathology and Laboratory Medicine, Medical University of South Carolina, Charleston, SC 29425, USA;; 2 Department of Histology and Embryology, University of South China, Hengyang City, Hunan Province 421001, P.R. China

**Keywords:** resveratrol, ionizing radiation, non-small cell lung cancer, DNA damage, premature senescence, reactive oxygen species

## Abstract

Radiotherapy is used in >50% of patients during the course of cancer treatment both as a curative modality and for palliation. However, radioresistance is a major obstacle to the success of radiation therapy and contributes significantly to tumor recurrence and treatment failure, highlighting the need for the development of novel radiosensitizers that can be used to overcome tumor radioresistance and, thus, improve the efficacy of radiotherapy. Previous studies indicated that resveratrol (RV) may sensitize tumor cells to chemotherapy and ionizing radiation (IR). However, the mechanisms by which RV increases the radiation sensitivity of cancer cells have not been well characterized. Here, we show that RV treatment enhances IR-induced cell killing in non-small cell lung cancer (NSCLC) cells through an apoptosis-independent mechanism. Further studies revealed that the percentage of senescence-associated β-galactosidase (SA-β-gal)-positive senescent cells was markedly higher in cells treated with IR in combination with RV compared with cells treated either with IR or RV alone, suggesting that RV treatment enhances IR-induced premature senescence in lung cancer cells. Comet assays demonstrate that RV and IR combined treatment causes more DNA double-strand breaks (DSBs) than IR or RV treatment alone. DCF-DA staining and flow cytometric analyses demonstrate that RV and IR combined treatment leads to a significant increase in ROS production in irradiated NSCLC cells. Furthermore, our investigation show that inhibition of ROS production by N-acetyl-cysteine attenuates RV-induced radiosensitization in lung cancer cells. Collectively, these results demonstrate that RV-induced radiosensitization is associated with significant increase of ROS production, DNA-DSBs and senescence induction in irradiated NSCLC cells, suggesting that RV treatment may sensitize lung cancer cells to radiotherapy via enhancing IR-induced premature senescence.

## Introduction

Radiotherapy is used in >50% of patients during the course of cancer treatment both as a curative modality and for palliation (1). However, radioresistance is a major obstacle to the success of radiation therapy and contributes significantly to tumor relapse and treatment failure. Therefore, there is a critical need for the development of novel radiosensitizers that can be used clinically to overcome tumor radioresistance and thus improve the efficacy of radiotherapy. Resveratrol (RV) is a small molecule natural component of grapes and red wine that has been shown to be a potential anticancer and chemopreventive agent (2–5). Interestingly, recent studies have indicated that treatment with RV can sensitize tumor cells to chemotherapeutic agents and IR induced cell death (6–9). However, the mechanisms by which RV increases the radiation sensitivity of cancer cells remain to be determined.

Cellular senescence is characterized as an irreversible cell cycle arrest that can be triggered by many types of intrinsic and extrinsic stresses, including IR (10–12). Senescence limits the life span and proliferative capacity of cells, therefore the induction of senescence is regarded as an important mechanism of cancer prevention (13,14). Notably, it has been suggested that therapy-induced senescence is an important mechanism through which many anticancer agents including IR suppresses tumor cell growth (15). Moreover, our recent studies have revealed that IR-induced tumor cell killing is largely attributed to the induction of senescence but not apoptosis in lung cancer cells, suggesting that the induction of premature senescence may play a pivotal role in mediating the anticancer effects of radiation therapy (16). Furthermore, we and others have demonstrated that RV is a potent small molecule inducer of cellular senescence (17,18). However, it is largely unknown if and how RV treatment may affect IR-induced premature senescence in tumor cells. Therefore, the goal of this study was to determine if RV treatment could sensitize lung cancer cells to radiotherapy by increasing IR-induced premature senescence. Here, we show that RV treatment enhances IR-induced cell killing in NSCLC cells through an apoptosis-independent mechanism. Our subsequent investigations further demonstrate that the radiosensitizing effect of RV is associated with increased DNA-DSBs and SA-β-gal staining in irradiated NSCLC cells, suggesting that RV treatment may sensitize lung cancer cells to radiotherapy by enhancing IR-induced senescence.

## Materials and methods

### Reagents

Resveratrol (trans-3,4′,5-trihydroxystilbene) and all other chemicals were purchased from Sigma (St. Louis, MO, USA). Dulbecco’s modified Eagle’s medium (DMEM) and other culture media were obtained from Invitrogen (Carlsbad, CA, USA). Rabbit anti-human phospho-p53, phospho-Akt, phospho-chk2 and phospho-mTOR monoclonal antibodies along with total Akt, chk2 and mTOR antibodies were purchased from Cell Signaling (Danvers, MA, USA). Mouse anti-human p53 and p21 monoclonal antibodies were obtained from Santa Cruz Biotechnology. Monoclonal β-actin antibody was purchased from Sigma. Senescence-associated β-galactosidase (SA-β-gal) staining kit was purchased from Cell Signaling.

### Cell lines and culture

Human non-small cell lung cancer (NSCLC) cell lines A549 and H460 were purchased from American Type Culture Collection. A549 cells were cultured in DMEM medium containing 10% FBS, 2 mM L-glutamine and 100 μg/ml of penicillin-streptomycin (Invitrogen). H460 cells were grown in RPMI-1640 medium containing 10% FBS, 2 mM L-glutamine and 100 μg/ml of penicillin-streptomycin (Invitrogen).

### Clonogenic survival assay

Clonogenic assays were performed to determine the effects of RV treatment on IR-induced cell death in NSCLC cells. Briefly, cells were seeded in 60-mm dishes at appropriate densities in triplicate and exposed to different doses (0–8 Gy) of irradiation at 4 h after RV pre-treatment using a ^137^Cs irradiator (J.L. Shepherd and Associates, Glendale, CA, USA) at a rate of 2.1 Gy/min. Twenty-four hours after irradiation, culture media were replaced with fresh complete media to remove drug and followed by incubation at 37°C for 8–12 days to allow the formation of colonies. Colonies were fixed and stained with 0.5% crystal violet (Sigma) in methanol for 30 min. The number of colonies (≥50 cells) was scored using a microscope. The surviving fraction was calculated as the ratio of the plating efficiency of the treated cells to that of control cells. The dose enhancement ratio (DER) was calculated as the dose (Gy) of radiation that yielded a surviving fraction of 0.3 for control divided by that for the RV treated cells.

### Senescence-associated β-galactosidase (SA-β-gal) staining

*In situ* staining of SA-β-gal was performed to determine the senescent cells in irradiated NSCLC cells using a senescence β-galactosidase staining kit (Cell Signaling) as we previously reported (18,19).

### Comet assay

Neutral comet assay was employed to determine DNA-DSBs in irradiated NSCLC cells by using a Comet Assay^®^ kit (Trevigen, Gaithersburg, MD, USA) according to the manufacturer’s instructions. Briefly, cells were mixed with Comet Assay™ low-melting agarose at a ratio of 1:10 (v/v) and spread evenly on slides. The cells were treated with CometAssay lysis solution at 4°C for 1 h, submerged in cold neutral electrophoresis buffer and subjected to electrophoresis at 21 V for 30 min. The cells were stained with SYBR^®^ Green I and viewed using a Zeiss Axio Observer Z1 microscope. The images were captured and processed using the AxioVision (4.7.1.0) software (Carl Zeiss). The percentage of DNA tail moment were evaluated with the TriTek Comet Score^TM^ software (Version 1.5.2.6; TriTek Corp., VA, USA).

### Western blot analysis

Protein samples were extracted using cell lysis buffer (Cell Signaling) supplemented with a cocktail of proteinase inhibitors (Sigma). The protein concentrations were quantified using the Bio-Rad Dc protein assay kit (Bio-Rad Laboratories, Hercules, CA, USA). Western blot analysis was performed as previously described (18). Briefly, 50 μg of protein samples were resolved on 10% Mini-Protean TGX gels (Bio-Rad) and transferred onto 0.2 μm PVDF membrane (Millipore). Blots were blocked with 5% non-fat milk for 1-2 h at room temperature and then probed with primary antibodies and incubated at 4°C overnight. After extensive washing with TBS-T, blots were incubated with appropriate HRP-conjugated secondary antibody for 1 h at room temperature. Protein bands were detected using an ECL Plus Western Blotting Detection System (GE Healthcare Life Science).

### Flow cytometric analysis of ROS

Intracellular levels of ROS were measured by flow cytometric analysis as we previously reported (20). Briefly, cells were loaded with 5 μM of 2′,7′-dichlorodihydrofluorescein diacetate (DCF-DA) and incubated at 37°C for 30 min. The levels of ROS in NSCLC cells were analyzed by measuring the mean fluorescence intensity (MFI) of DCF using a FACSCalibur flow cytometer (Becton-Dickinson, San Jose, CA, USA).

### Statistical analysis

All experiments were repeated independently at least three times. Paired comparisons were carried out using Student’s t-test. Multiple group comparisons were performed using analysis of variance (ANOVA). Differences were considered statistically significant at p<0.05. All analyses were carried out with the GraphPad Prism program (GraphPad Software, Inc. San Diego, CA, USA).

## Results

### RV enhances IR-induced cell killing in lung cancer cells via an apoptosis-independent mechanism

**Figure 1.**
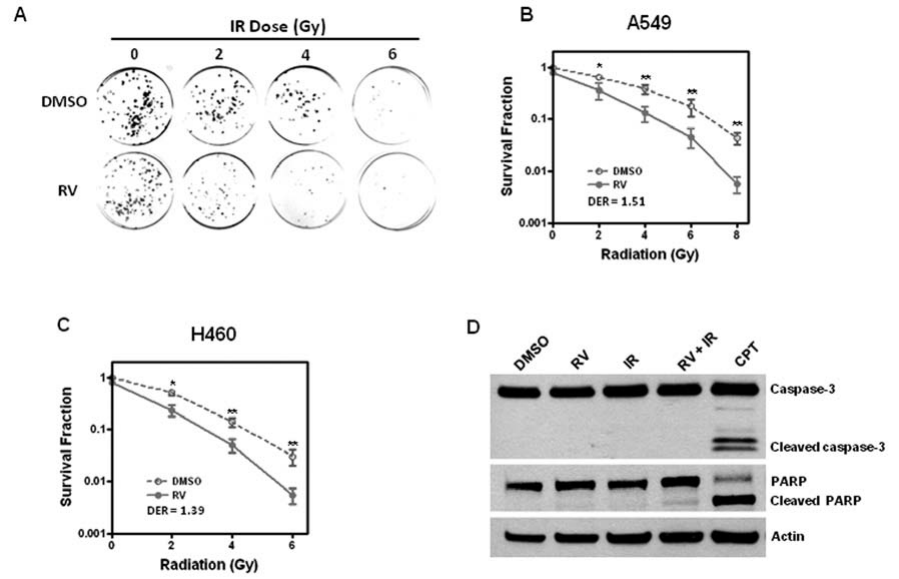
RV sensitizes lung cancer cells to IR-induced tumor cell killing. (A) Representative images of clonogenic assays showing that IR inhibits the colony-forming capacity of cancer cells in a dose-dependent manner and that RV enhances the tumor suppressive effect of IR. (B) Clonogenic assays indicate that RV sensitizes A549 to IR-induced cell killing. (C) Clonogenic assays show that RV sensitizes H460 to IR-induced cell killing. (D) Western blot analyses were performed to determine the expression levels of cleaved caspase-3 and cleaved PARP in H460 cells. Actin was probed as a loading control. ^*^p<0.05 vs. DMSO control; ^**^p<0.01 vs. DMSO control.

Previous studies showed that RV treatment increased the sensitivity of tumor cells to chemotherapy and IR induced cell death (6–9). Here, we sought to investigate whether RV treatment could sensitize NSCLC cells to IR-induced cell killing. To this end, A549 and H460 cells were pre-incubated with RV (20 μM) or DMSO as a vehicle control for 4 h prior to exposure to different doses of IR treatment. Then clonogenic assays were performed to determine if RV treatment has any impact on IR-induced tumor cell killing. The results show that preincubation with RV significantly enhances the cell killing effects of IR with a DER of 1.51 for A549 cells and 1.39 for H460 cells (Fig. 1A–C), suggesting that RV is a potential radiosensitizer that can increase the sensitivity of lung cancer cells to IR-induced cell killing.

To further explore the mechanisms by which RV increases the radiation sensitivity of lung cancer cells, we investigated whether RV could promote IR-induced apoptosis. Because activated caspase-3 and cleaved PARP are well-documented measurements of apoptosis (21,22), we examined whether RV treatment affects the expression of activated caspase-3 and cleaved PARP in irradiated NSCLC cells. Western blot analyses show that RV treatment has no significant effect on the expression of cleaved PARP and activated caspase-3 in H460 cells regardless of IR treatment. In contrast, camptothecin (CPT) treatment results in a pronounced increase in the expression levels of cleaved PARP and activated caspase-3 (Fig. 1D). These results demonstrate for the first time that RV enhances IR-induced tumor cell killing via an apoptosis-independent mechanism.

### RV promotes IR-induced premature senescence in lung cancer cells

**Figure 2.**
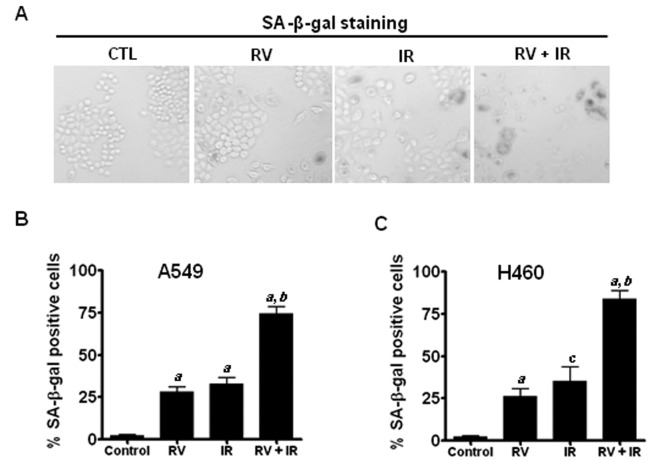
Treatment with RV increases IR-induced premature senescence in NSCLC cells. (A) SA-β-gal staining assays were performed to determine senescent cells in irradiated lung cancer cells. Representative microimages of SA-β-gal staining in H460 cells are presented. (B) SA-β-gal assays show that RV pretreatment increases IR-induced premature senescence in A549 cells. (C) SA-β-gal assays show that RV pretreatment augments IR-induced premature senescence in H460 cells. ^a^p<0.01 vs. control; ^b^p<0.01 vs. IR; ^c^p<0.05 vs. control.

Our recent studies have shown that IR induces premature senescence in lung cancer cells in a dose-dependent manner, suggesting that the induction of senescence plays an important role in IR-induced tumor suppression (16). However, it remains to be determined whether RV radiosensitizes lung cancer cells by augmenting IR-induced premature senescence. To this end, SA-β-gal staining was employed to detect senescent cells in irradiated A549 and H460 cells with or without RV treatment. The results show that RV and IR combined treatment induces more SA-β-gal positive senescent cells than either RV or IR treatment alone in NSCLC cells (Fig. 2). These results suggest that RV may radiosensitize lung cancer cells by enhancing IR-induced premature senescence.

### RV treatment increases IR-induced DNA-DSBs in NSCLC cells

**Figure 3.**
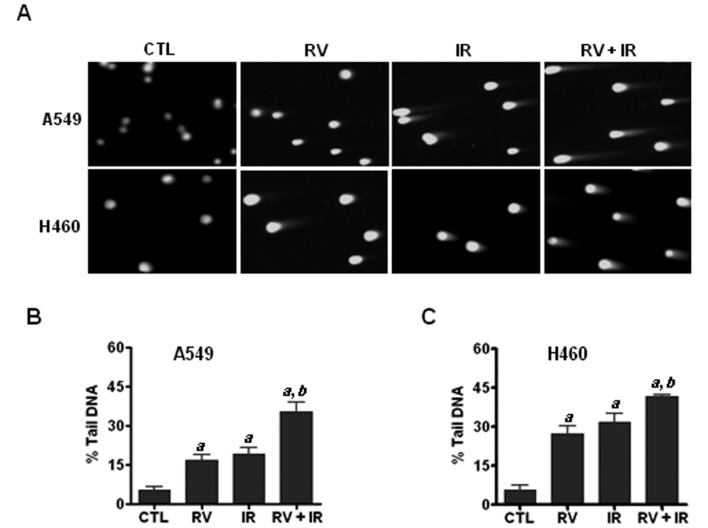
Treatment with RV enhances IR-induced DNA damage in NSCLC cells. (A) Representative photomicrographs of comet assays showing that IR-induced DNA-DSBs in A549 (upper panel) and H460 cells (lower panel), respectively. (B) The percentage of tail DNA in A549 cells with different treatments was quantified. (C) The percentage of tail DNA in H460 cells with different treatments was quantified. ^a^p<0.001 vs. control; ^b^p<0.05 vs. IR.

Next we asked the question as to how RV enhances IR-induced premature senescence in lung cancer cells. Given that DNA damage is a major cause underlying chemotherapy and ionizing radiation induced premature senescence (18,20,23), we hypothesized that RV treatment may enhance IR-induced senescence via increasing DNA damage in irradiated NSCLC cells. To test this hypothesis, we performed neutral comet assays to measure DNA-DSBs in irradiated NSCLC cells with or without RV treatment. The results demonstrate that RV and IR combined treatment results in more DNA-DSBs than either IR or RV treatment alone (Fig. 3). These results strongly support the hypothesis that RV treatment may promote the induction of senescence in irradiated lung cancer cells by increasing IR-induced DNA damage.

### RV inhibits the phosphorylation of Akt and mTOR in lung cancer cells

**Figure 4.**
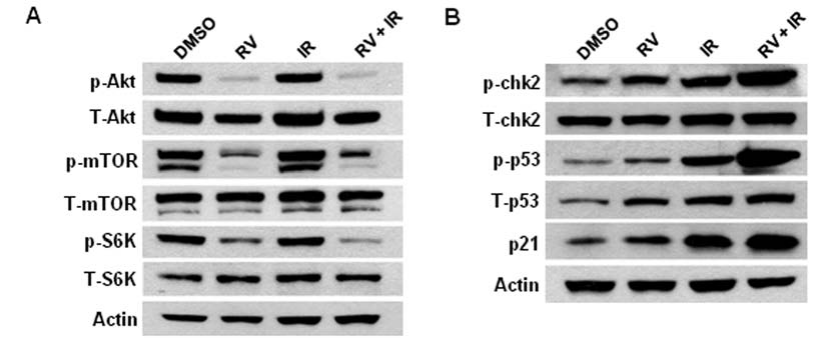
RV inhibits the phosphorylation of Akt and increases the accumulation of p-p53 and p-chk2. (A) Western blot analyses were performed to determine the expression levels of p-Akt, p-mTOR and p-S6K in H460 cell. (B) Western blot analyses were performed to determine the expression levels of DNA damage response protein p-chk2 and p-p53 in H460 cells. Actin was probed as a loading control.

It has been shown that the inhibition of Akt activity sensitizes tumor cells to anticancer therapy (24). However, it has yet to be determined if RV treatment affects the activities of Akt and mTOR in NSCLC cells. To address this issue, western blot analyses were performed to determine the expression levels of phosphorylated Akt (p-Akt, Ser473) and phosphorylated mTOR (p-mTOR, Ser2448) in lung cancer cells. The results show that RV treatment significant inhibits the expression levels of p-Akt and p-mTOR in irradiated H460 lung cancer cells (Fig. 4A). Moreover, our studies also show that RV treatment results in a significant decline in the phosphorylation of p70 S6 kinase (p-S6K, T389), a downstream target of mTOR. Furthermore, we show that RV and IR combined treatment leads to marked increases of phosphorylated p53 (p-p53, Ser15) and phosphorylated chk2 (p-chk2, T78) levels in irradiated NSCLC cells than in cells treated with IR or RV alone (Fig. 4B). Because phosphorylation of p53 and chk2 are important biomarkers of DNA damage, these results further confirm that RV treatment enhances IR-induced DNA damage in NSCLC cells.

### RV treatment increases ROS production in irradiated lung cancer cells

**Figure 5.**
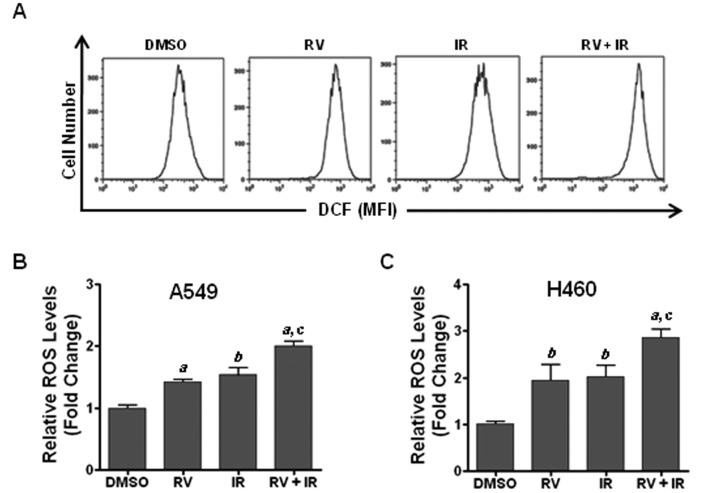
Effect of RV treatment on IR-induced ROS generation in lung cancer cells. (A) The levels of ROS were measured by DCF-DA staining and flow cytometric analyses at 24 h after 5 Gy of irradiation. Shown is a representative analysis of ROS production in lung cancer cells by flow cytometry. (B) The relative levels of ROS in A549 cells are presented as fold change compared to control cells. (C) The relative levels of ROS in H460 cells are shown as fold change compared to control cells. ^a^p<0.01 vs. control; ^b^p<0.05 vs. control; ^c^p<0.05 vs. IR.

Previous studies have shown that ROS play a critical role in modulating genotoxic stress-induced DNA damage and that DNA damage is able to induce premature senescence in tumor cells (16,20,23). However, it remains to be determined whether the generation of ROS is involved in mediating the radiosensitization effect of RV. To address this question, we examined the levels of ROS in lung cancer cells using DCF-DA staining along with flow cytometric analyses as previously described (20). The results show that RV treatment markedly increases ROS production in irradiated lung cancer cells compared with those cells treated with RV or IR alone (Fig. 5). These results suggest that increasing of ROS production may play a pivotal role in RV-induced radiosensitization.

### Inhibition of ROS by NAC attenuates the radiosensitizing effect of RV in lung cancer cells

**Figure 6.**
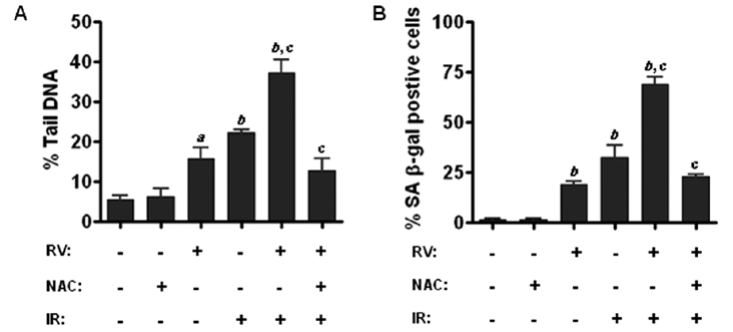
Treatment with NAC attenuates RV-induced radiosensitization in lung cancer cells. (A) H460 cells were pretreated with NAC prior to IR exposures. Two hours after IR, neutral comet assays were performed to assess DNA-DSBs in lung cancer cells. (B) SA-β-gal staining was employed to determine senescence in H460 cells. ^a^p<0.05 vs. control; ^b^p<0.01 vs control; ^c^p<0.01 vs IR.

To determine the role of ROS in RV-mediated radiosensitization, we sought to examine whether inhibition of ROS production by antioxidant NAC has any impact on RV-mediated enhancement of IR-induced DNA damage and premature senescence in lung cancer cells. Because comet assay is a well characterized approach to measure DNA-DSBs, we performed comet assays and found that RV and IR combined treatment results in more DNA damage in lung cancer cells than either IR or RV treatment alone (Fig. 6A). More importantly, our data further demonstrate that preincubation with NAC attenuates the enhancement of RV on IR-induced DNA-DSBs (Fig. 6A). Furthermore, SA-β-gal staining assays reveal that inhibition of ROS production by NAC markedly diminishes the enhancement effect of RV on IR-induced senescence in lung cancer cells (Fig. 6B). Together, these findings suggest that RV treatment may enhance IR-induced premature senescence via increasing ROS-mediated DNA damage in lung cancer cells.

## Discussion

Because of the high cost, debilitating side effects, and therapeutic limitations of conventional chemotherapy and radiotherapy, it is estimated that ∼40% of Americans use complementary and alternative medicine (CAM), including herbal medicine and natural products (NPs), for cancer prevention and treatment (4). The use of NPs as antitumor agents for the management of human cancers is an attractive idea because they are readily available and exhibit little or no toxicity. RV is such an NP that has been shown to exhibit both anticancer and chemopreventive potentials (4,25). A number of previous studies indicated that RV treatment may sensitize tumor cells to chemotherapeutic agents and radiation induced cell death (6–9). However, the mechanisms whereby RV sensitizes tumor cells to radiotherapy are poorly understood. In this report, we provide evidence demonstrating that RV treatment markedly increases DNA-DSBs and SA-β-gal staining in irradiated NSCLC cells, suggesting that RV may sensitize lung cancer cells to radiotherapy via enhancing IR-induced premature senescence.

It has been well documented that the induction of senescence is an important alternative mechanism underlying chemo- and radiotherapy-induced tumor suppression (11,12,15,16,18). In contrast to most of the previous studies in which high concentrations of RV were used to induce tumor cell apoptosis (26,27), in this study we utilized a relatively lower dose of RV to sensitize cancer cells to IR-induced senescence. The advantage of using low concentration of RV is that it could be clinically achievable (28,29). Notably, therapy-induced senescence can be achieved at much lower doses of chemotherapy than those required to induce apoptosis (15,18). Compared to the traditional apoptosis inducing strategies, this low dose approach may significantly reduce the side effects of cancer therapy and thus improve the quality of life for cancer patients.

Radioresistance is a major obstacle to successful treatment in lung cancer radiotherapy (30–32). The use of radiosensitizer holds the promise of overcoming radioresistance and thus improving treatment outcomes. A variety of kinase inhibitors such as Akt, mTOR, and chk1 inhibitors have been extensively investigated as potential radiosensitizers (33–37). However, the clinical benefits of these inhibitors to improve treatment outcomes are limited. In addition, there are growing concerns as to the potential toxicity and side effects of these kinase inhibitors because of their broad biological activities. Therefore, there is a critical need for the development of novel and more effective radiosensitizers to meet this challenge. In contrast to the kinase inhibitors, natural compounds such as RV have been presumed to be safer than synthetic compounds due to their presence in diet, wide availability and tolerability (25,38). In fact, it has been demonstrated in a phase I study that it is clinically safe to orally take ≤5 g of RV per day (39). Together, these studies support a further development of RV as a safer, affordable and effective radiosensitizer.

We and others have shown that ROS plays a critical role in mediating chemotherapy- and IR-induced DNA damage and cell killing (18,20,40,41). Therefore, we hypothesized that RV treatment may sensitize tumor cells to IR-induced premature senescence via increasing ROS-mediated DNA damage. In agreement with this hypothesis, we found that RV-induced enhancement on IR-induced DNA damage was associated with a significant increase in ROS generation in irradiated NSCLC cells (Fig. 5). Moreover, our studies also show that inhibition of ROS by NAC attenuates the sensitizing effects of RV on IR-induced DNA damage and premature senescence in lung cancer cells, suggesting that ROS may play an important role in mediating the radiosensitizing effect of RV (Fig. 6). Consistent with these observations, it was reported that treatment of bladder cancer cells with buthionine sulphoximine (BSO), a glutathione synthesis inhibitor, significantly increases ROS production and enhances cisplatin-induced cytotoxicity (42). These observations support the concept that modulating oxidative stress in tumor cells could be an effective therapeutic strategy for cancer treatment. Taken together, these results demonstrate for the first time that RV-induced radiosensitization is associated with marked increases in ROS production, DNA-DSBs, and senescence induction in irradiated NSCLC cells, suggesting that increasing the IR-induced premature senescence could be exploited as a novel strategy to sensitize lung cancer cells to radiotherapy.
